# Unravelling the Miscibility of Poly(2-oxazoline)s: A Novel Polymer Class for the Formulation of Amorphous Solid Dispersions

**DOI:** 10.3390/molecules25163587

**Published:** 2020-08-06

**Authors:** Melissa Everaerts, Ali Tigrine, Victor R. de la Rosa, Richard Hoogenboom, Peter Adriaensens, Christian Clasen, Guy Van den Mooter

**Affiliations:** 1 Drug Delivery and Disposition, Department of Pharmaceutical and Pharmacological Sciences, KU Leuven, 3000 Leuven, Belgium; melissa.everaerts@kuleuven.be; 2 Supramolecular Chemistry Group, Centre of Macromolecular Chemistry (CMaC), Department of Organic and Macromolecular Chemistry, Ghent University, Krijgslaan 281-S4, 9000 Ghent, Belgium; ali.tigrine@ugent.be (A.T.); victor.retamerodelarosa@ugent.be (V.R.d.l.R.); richard.hoogenboom@ugent.be (R.H.); 3 Applied and Analytical Chemistry Department, Institute for Materials Research, Hasselt University, 3590 Diepenbeek, Belgium; peter.adriaensens@uhasselt.be; 4 Department of Chemical Engineering, Soft Matter, Rheology and Technology, KU Leuven, 3001 Leuven, Belgium; christian.clasen@kuleuven.be

**Keywords:** amorphous solid dispersions, poly(2-oxazoline)s, miscibility, modulated differential scanning calorimetry, solid-state nuclear magnetic resonance spectroscopy

## Abstract

Water-soluble polymers are still the most popular carrier for the preparation of amorphous solid dispersions (ASDs). The advantage of this type of carrier is the fast drug release upon dissolution of the water-soluble polymer and thus the initial high degree of supersaturation of the poorly soluble drug. Nevertheless, the risk for precipitation due to fast drug release is a phenomenon that is frequently observed. In this work, we present an alternative carrier system for ASDs where a water-soluble and water-insoluble carrier are combined to delay the drug release and thus prevent this onset of precipitation. Poly(2-alkyl-2-oxazoline)s were selected as a polymer platform since the solution properties of this polymer class depend on the length of the alkyl sidechain. Poly(2-ethyl-2-oxazoline) (PEtOx) behaves as a water-soluble polymer at body temperature, while poly(2-*n*-propyl-2-oxazoline) (PPrOx) and poly(2-*sec*-butyl-2-oxazoline) (PsecBuOx) are insoluble at body temperature. Since little was known about the polymer’s miscibility behaviour and especially on how the presence of a poorly-water soluble drug impacted their miscibility, a preformulation study was performed. Formulations were investigated with X-ray powder diffraction, differential scanning calorimetry (DSC) and solid-state nuclear magnetic resonance spectroscopy. PEtOx/PPrOx appeared to form an immiscible blend based on DSC and this was even more pronounced after heating. The six drugs that were tested in this work did not show any preference for one of the two phases. PEtOx/PsecBuOx on the other hand appeared to be miscible forming a homogeneous blend between the two polymers and the drugs.

## 1. Introduction

Active pharmaceutical ingredients (APIs) with a poor aqueous solubility and low dissolution rate are a well-known and frequently encountered phenomenon. A popular approach to ensure adequate bioavailability after oral administration of a poorly water-soluble drug is to formulate the drug with an inert carrier in which the drug is molecularly dispersed, which is better-known as an amorphous solid dispersion (ASD). In an ASD, there is no long-range ordering of drug molecules like in the crystalline state, leading to a higher apparent solubility of the drug [1]. The inert carrier, which is often a water-soluble polymer, such as hydroxypropyl methylcellulose, polyvinylpyrrolidone or polyvinylpyrrolidone vinyl acetate [2,3,4], serves as a physical barrier to prevent the onset of crystallization and thus increases the stability of the formulation during storage and dissolution.

One of the advantages of a water-soluble polymeric carrier is that it can also act as a precipitation inhibitor during drug release in the gastrointestinal (GI) tract [5,6]. Next to that, a rapid drug release is often ensured as the water-soluble polymer will dissolve almost instantaneously in the GI fluids, making the drug molecules easily available for absorption. However, rapid drug release has also been associated with a higher risk for precipitation as this burst release effect will generate a high degree of supersaturation leading to either precipitation if the amorphous solubility is exceeded [7] or precipitation of crystalline material due to the formation of nuclei, followed by crystal growth [8]. To delay this burst release, researchers have started to investigate insoluble carriers for the formulation of ASDs. As the carrier will not dissolve, drug release is based on diffusion, creating a more steady increase in drug concentration and thus reducing the risk of precipitation [8]. Since drug release is based on diffusion, initial porosity of the formulation, possible changes in this property and the formation of physical crosslinks can affect the drug release and can, therefore, lead to insufficient drug release as previously demonstrated by our research group [9].

**Figure 1:**
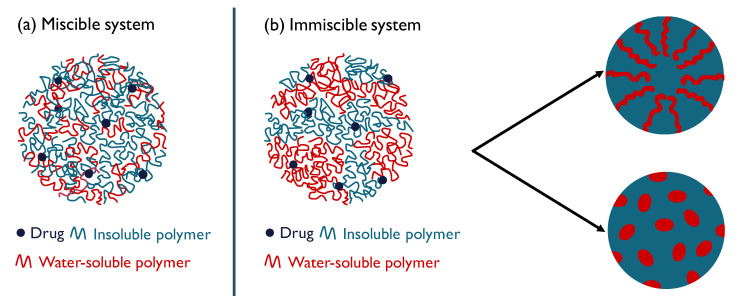
Schematic representation of an amorphous solid dispersion (ASD) particle based on a miscible system (**a**) and an immiscible system (**b**). In the case of an immiscible system, distribution of the water-soluble polymer in the ASD particle will determine what kind of pores that will be formed and hence is expected to influence the drug release from this particle.

In 2018, Lugtu-Pe et al. [10] reported on a controlled release ASD where a combination of polyvinylpyrrolidone (PVP) as a water-soluble polymer and polyvinyl acetate as a water-insoluble polymer were used as an inert carrier system. Conjoining a water-soluble and insoluble polymer could be a promising formulation strategy for ASDs as the insoluble carrier can act as a drug-retarding agent while the water-soluble fraction can create pores upon dissolution, providing adequate diffusion and release of drug molecules. However, this novel combination of ternary ASDs has not yet been extensively investigated and, especially, no studies appeared on how the miscibility behaviour of the two polymers can affect the drug release. In the case of a miscible system, the drug molecules will be distributed in a homogeneous polymer system of the insoluble and water-soluble polymer (Figure 1a). In an immiscible system on the other hand, discrete regions of both polymer fractions will be present (Figure 1b). The drug can then either have a preference for one of the two phases or be equally distributed over both regions. Next to that, different distributions of water-soluble polymers are also expected to have an impact on the drug release as they will lead to a different distribution of pores after their dissolution.

**Figure 2:**
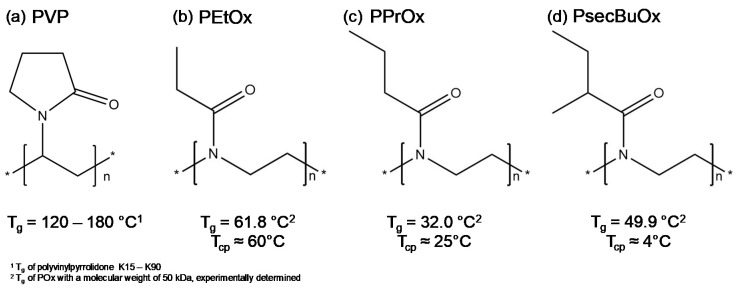
Chemical structure and corresponding glass transition temperature (T_g_) of polyvinylpyrrolidone (**a**) [22], poly(2-ethyl-2-oxazoline) (**b**), poly(2-*n*-propyl-2-oxazoline) (**c**) and poly(2-*sec*-butyl-2-oxazoline) (**d**). Created with ChemDraw Professional software (PerkinElmer). For poly(2-alkyl-2-oxazolines), cloud point temperatures (T_cp_) are also given [18].

An interesting polymer class to further explore the potential of combining a water-soluble and insoluble carrier in an ASD are the poly(2-alkyl-2-oxazoline)s (PAOx). The use of this polymer class in the field of ASDs has been reported by Claeys et al. [11], Policianova et al. [12], Fael et al. [13], Ruiz-Rubio et al. [14], Abilova et al. [15], Moustafine et al. [16] and Boel et al. [17]. One of the most important advantages of this polymer class is that its physicochemical properties such as glass transition temperature (T_g_), solubility and mechanical strength can easily be altered by adjusting the side chain of the 2-oxazoline monomer or by end-group functionalization [18,19,20]. In addition, its structure is very similar to that of PVP, a water-soluble polymer commonly used in ASDs, albeit having a significantly lower T_g_ (Figure 2) [21].

In the current study, a combination of poly(2-ethyl-2-oxazoline) (PEtOx) with either poly(2-*n*-propyl-2-oxazoline) (PPrOx) or poly(2-*sec*-butyl-2-oxazoline) (PsecBuOx; racemic mixture of *sec*-butyl side chains) was selected as a carrier for ASDs. All three polymers are thermoresponsive and are water-soluble at low temperatures, but phase-separate upon heating. Since PEtOx has a cloud point temperature (T_cp_) of 60 °C and is water-soluble below this temperature [19], it will serve as a water-soluble carrier in the ASD. PPrOx and PsecBuOx on the other hand have a T_cp_ located around 25 °C and 4 °C respectively, meaning that both polymers will be water-insoluble at body temperature [19]. Very recently we reported that PEtOx and PPrOx with a number of the average molecular weight above 10 kg/mol are immiscible and form phase separated blends after film casting or electrospinning [23]. To the best of our knowledge, there is no information on the miscibility behaviour of the three polymers in the presence of an API. Since it is of utmost importance to understand the miscibility behaviour of a polymer blend in order to allow a rational selection of a suitable carrier for the formulation of an ASD, a preformulation study was performed. In this preformulation study, the goal was to assess the solid state behaviour of these ASDs and in the case of immiscibility of the polymers, whether the investigated model drugs, indomethacin (IND), itraconazole (ITZ), fenofibrate (FEN), miconazole (MIC), ibuprofen (IBU) and etravirine (ETR) had a preference for one of the two phases. Our hypothesis was that if the drug would not have a preference for one of the two phases, both T_g_s would shift equally towards the T_g_ of the drug and thus the ratio of the two T_g_s would remain the same as for the polymer blend without API. It should be pointed that the focus of current work was on assessing the miscibility behaviour of these ASDs and not on their respective pharmaceutical performance.

Whether or not a polymer blend is miscible, depends not only on specific interactions such as ion-ion, ion-dipole, dipole-dipole and hydrogen bonds, but also on the temperature at which the polymer blend is processed and stored [24]. Blends might demonstrate lower or upper critical solution temperature behaviour [24]. In order to assess the effect of the temperature at which the blends were processed, different process conditions and manufacturing techniques were investigated for both the PEtOx/PPrOx and the PEtOx/PsecBuOx blends. In the current work, both blends were spray dried at two different inlet temperatures, namely 35 and 45 °C and electrosprayed at 25 °C to investigate the effect of the temperature and process method on the miscibility of both PEtOx/PPrOx and PEtOx/PsecBuOx blends. It should be noted that the droplet formation process differs between spray drying and electrospraying [25]. During spray drying droplets are formed by applying a mechanical force on a liquid stream, while with electrospraying droplets are created by the generation of electric charges. For electrospraying, this results in the formation of a jet that breaks down into small droplets, which leads to an efficient evaporation process, even at temperatures below the boiling point of the solvent [26]. Droplets that are created via spray drying on the other hand, are generally larger compared to those obtained with electrospraying and a higher processing temperature will be required to ensure adequate evaporation of the solvent [25,26].

## 2. Results and Discussion

### 2.1. Solid State Analysis of Pure Polymers: Starting Material Versus Spray Dried Material

**Figure 3:**
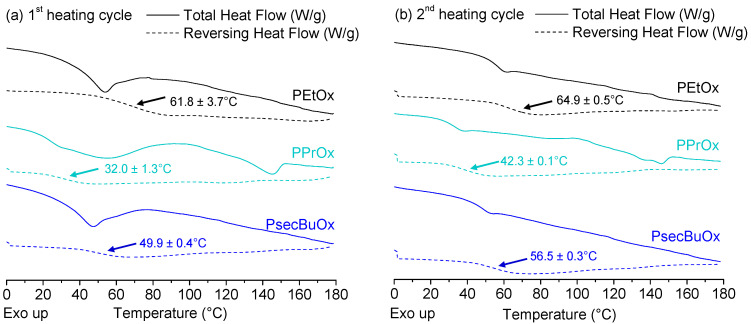
mDSC thermograms of the first (**a**) and second (**b**) heating cycle of PEtOx (black), PPrOx (cyan) and PsecBuOx (blue). Total heat flow (full line) and reversing heat flow (dashed line) are shown. Glass transitions are indicated with arrows in the reversing heat flow and the average glass transition temperature (*n* = 3) is given for each PAOx. Created with Origin 8.5 from OriginLab (Northampton, MA, USA).

In Figure 3 modulated differential scanning calorimetry (mDSC) thermograms of the first (a) and second heating cycle (b) are shown for unprocessed PEtOx, PPrOx and PsecBuOx together with their average T_g_ (*n* = 3). For both PEtOx and PsecBuOx, a sharp endothermic event due to enthalpic recovery can be observed in the total heat flow of the first heating cycle around their respective glass transitions. For PPrOx no sharp event can be observed, but a rather broad endothermic signal in the total heat flow was detected in the first heating cycle over the range of approximately 20–80 °C, which indicated that there was some residual solvent present in the raw starting material, causing a decrease in the T_g_ due to plasticizing effects. This was followed by an exothermic event, indicating the formation of crystalline regions and around 130 °C a broad melting endotherm was present for PPrOx. The observation of crystallization for PPrOx in the first heating cycle was not expected since previous articles reported the polymer to be fully amorphous [27,28]. A possible explanation for this unanticipated phenomenon is thermal history of the sample that may have led to a change in the conformational change in the polymer backbone inducing crystallinity. This effect has already been described by Katsumoto et al. [29] for poly(2-isopropyl-2-oxazoline), which crystallized irreversibly when an aqueous solution was heated above the lower critical solution temperature, which was ascribed to a conformational change of the polymer backbone. In the second heating cycle, two distinct melting endotherms could be observed for the pure PPrOx, meaning that the polymer demonstrated semicrystalline behaviour. Further investigations into the observed crystallinity of PPrOx were beyond the scope of this work, but will be included in our future studies.

**Figure 4:**
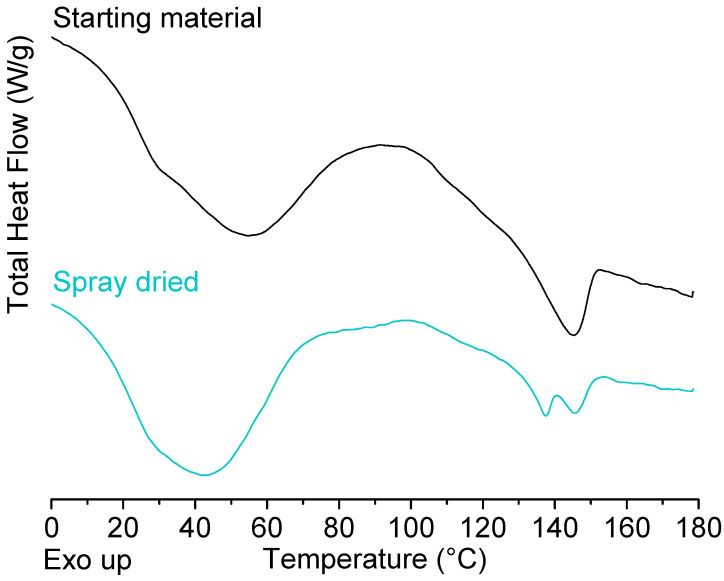
mDSC thermograms of the first heating cycle of PPrOx where the starting material (black) is compared to the spray dried material (cyan). Created with Origin 8.5 from OriginLab (Northampton, MA, USA).

Similar T_g_ values were found for the spray dried samples (see Supplementary Materials S1). It should be noted that for spray dried PEtOx, an additional isothermal segment was executed prior to the start of the measurement in order to remove the high amount of residual solvent (1.85%) of which the evaporation coincided with the glass transition and therefore led to a high standard deviation if the sample was not preheated at 40 °C for 30 min. In addition, two more distinct melting peaks could already be detected for spray dried PPrOx during the first heating cycle (Figure 4). Since this was not the case for the first heating cycle of the unprocessed PPrOx, where a broad melting peak over the entire temperature region was observed, it could be concluded that the two polymer fractions responsible for the melting endotherms were more separated in the spray dried PPrOx.

**Figure 5:**
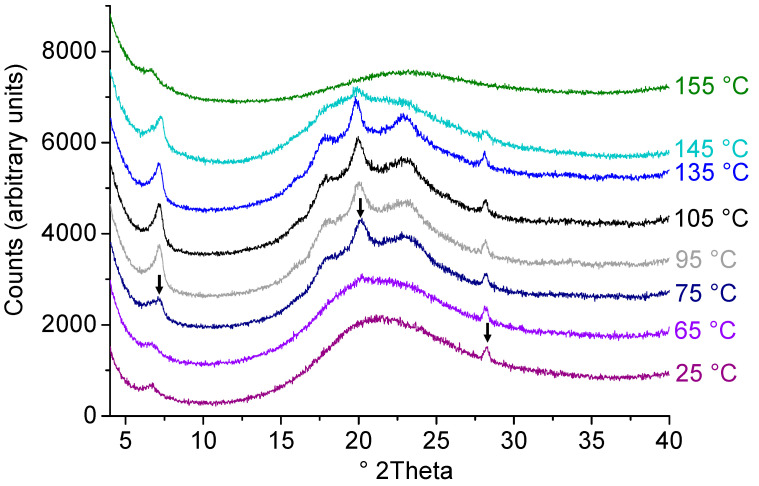
X-ray powder diffraction (XRPD) diffractograms of spray dried PPrOx, recorded during the temperature resolved experiment. Bragg peaks that were initially present or appeared upon heating are indicated with arrows. Created with Origin 8.5 from OriginLab (Northampton, MA, USA).

The semicrystalline behaviour of PPrOx was confirmed and further investigated with temperature resolved X-ray diffraction (XRD) of the spray dried powder (Figure 5) where a Bragg-peak around 28° 2theta was detected at 25 °C. Upon heating, two additional Bragg peaks appeared at 75 °C and at 155 °C all three Bragg peaks disappeared, indicating the further development of crystalline regions upon heating, followed by melting. In the past, Rettler at al. [30] already reported semicrystallinity of PPrOx, detected via Fourier-transform infrared spectroscopy albeit it was not observed by DSC.

### 2.2. Solid State Analysis of Polymer Mixtures

Since little was known about the miscibility behaviour of the different PAOx, besides our recent report on phase separation of PEtOx-PPrOx blends [23], formulations containing only PAOx were prepared through spray drying or film casting in order to assess the miscibility of both PAOx combinations in the absence of API. Various authors have already highlighted the importance of understanding the miscibility and phase behaviour of a binary carrier mixture in order to fully and correctly understand that of the ternary dispersions with API [31,32,33]. Next to that, addition of the API, a third component, could affect the miscibility behaviour of the two polymers.

#### 2.2.1. Poly(2-ethyl-2-oxazoline) and Poly(2-n-propyl-2-oxazoline)

**Figure 6:**
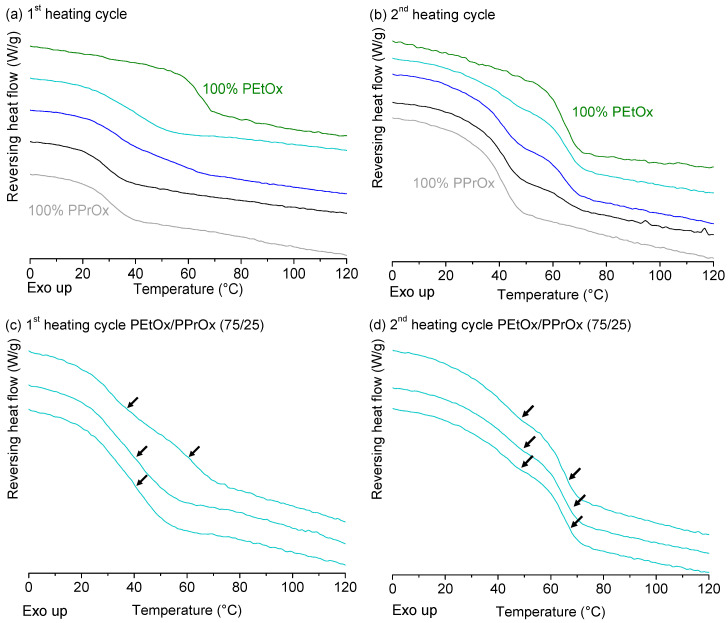
First (**a**) and second (**b**) heating cycle of spray dried PEtOx (green), PEtOx/PPrOx 3/1 (*m*/*m*) (cyan), PEtOx/PPrOx 1/1 (*m*/*m*) (blue), PEtOx/PPrOX 1/3 (*m*/*m*) (black) and PPrOx (grey). The three measurements that were performed for the blend consisting of 75% PEtOx and 25% PPrOx (% *m*/*m*) are displayed in (**c**,**d**). There was clearly inhomogeneity in the sample as demonstrated in the first heating cycle (**c**). In the second heating cycle (**d**) all three measurements showed a similar mDSC thermogram with two T_g_s at the same positions for all three measurements. Average T_g_ values (*n* = 3) can be found in Supplementary Materials S2a. Created with Origin 8.5 from OriginLab (Northampton, MA, USA).

Three different ratios of water-soluble (PEtOx) and water-insoluble (PPrOx) polymers were spray dried and analysed by mDSC (Figure 6a,b): 3/1, 1/1 and 1/3 (*m*/*m*). In the first heating cycle, the blends containing 25 and 75% PEtOx seemed to have a single T_g_, while the formulation made up from 50% PEtOx already demonstrated immiscibility during this first heating cycle as two T_g_s can be observed at 32.9 (±0.1) and 57.7 (±1.9) °C. However, the formulation consisting of 75% PEtOx, also turned out to be a heterogeneous polymer blend as two out of the three thermograms of the first heating cycle showed one broad T_g_, while two T_g_s were always present in the third thermogram (Figure 6c). Broad glass transitions can be associated with unfavourable mixing behaviour of the polymers [34] and this observation, together with the heterogeneity of the sample, is already a strong indication that this is a thermodynamically unstable system. The results of the second heating cycle further support this statement since now all three combinations have two T_g_s and were therefore considered immiscible (Figure 6d). For 3/1, 1/1 and 1/3 polymer blends of PEtOx and PPrOx, the following T_g_s were observed in the second heating cycle: 41.5 (±2.0) and 64.7 (±1.3) °C, 43.8 (±0.9) and 63.2 (±0.5) °C and 41.5 (±1.0) and 64.4 (±2.9) °C. For all three polymer blends, these values were situated close to the T_g_s of the homopolymers, indicating efficient phase separation. The fact that the immiscibility is even more apparent in the second heating cycle means that manufacturing processes such as hot melt extrusion (HME) will have an impact on the phase behaviour of this polymer blend. In this and other fusion-based processes, polymers are often heated to temperatures above their T_g_ [35]. Based on the effect of heating we observed during the mDSC measurements, it is expected that a higher processing temperature will lead to a more phase separated PEtOx/PPrOx system, not only for HME, but for all manufacturing processes where the blend is subjected to elevated temperatures.

Despite the fact that two T_g_s in a thermogram are a typical feature for immiscibility, different authors have already addressed the issue where a miscible polymer blend demonstrated two T_g_s when analysed with mDSC [36,37]. One of the possible explanations for this phenomenon is the occurrence of mesoscale composition fluctuations in the miscible polymer blend that lead to compositional heterogeneity [36]. For this reason solid-state (ss) ^1^H-wideline nuclear magnetic resonance (NMR) relaxation experiments were accomplished to determine the phase morphology via the T_1H_ and T_1ρH_ relaxation behaviour of the pure spray dried polymers and the blend (PEtOx/PPrOx; 1/1; Table 1). In chemistry, NMR relaxometry of nuclear spins is a well-known method used to describe the phase morphology of blends [38,39,40]. However, it was not possible to draw any conclusions about the miscibility of the 1-1 polymer blend based on ssNMR as the relaxation times of pure PEtOx and PPrOx were situated too close to each other. We can therefore only conclude that, based on mDSC, two T_g_s could be detected and that this is a strong indication that PEtOx-PPrOx forms an immiscible system. In our previous work we also visualized and confirmed the phase separation through fluorescence microscopy after labelling one of the polymers [23].

#### 2.2.2. Poly(2-ethyl-2-oxazoline) and Poly(2-sec-butyl-2-oxazoline)

**Figure 7:**
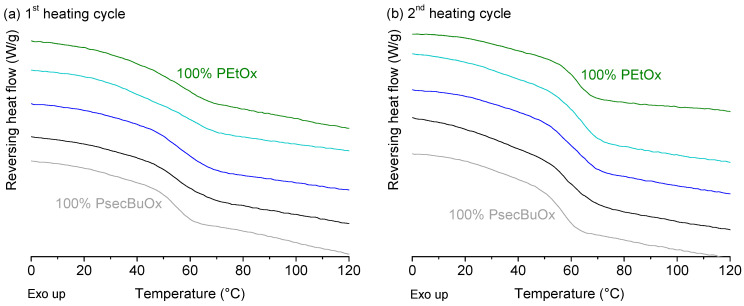
First (**a**) and second (**b**) heating cycle of film-casted PEtOx (green), PEtOx/PsecBuOx 2/1 (*m*/*m*; cyan), PEtOx/PsecBuOx 1/1 (*m*/*m*; blue), PEtOx/PsecBuOx 1/2 (*m*/*m*; black) and PsecBuOx (grey). The T_g_s of pure PEtOx and PsecBuOx are in each other’s vicinity and thus make it difficult to draw conclusions concerning the miscibility, based on these thermograms. Average T_g_ values (*n* = 3) can be found in Supplementary Materials S2b. Created with Origin 8.5 from OriginLab (Northampton, MA, USA).

Thermograms of PEtOx-PsecBuOx 2/1, 1/1 and 1/2 (*m*/*m*) combinations, prepared by film casting, are shown in Figure 7. In both the first and second heating cycle, the three combinations appeared to have a single T_g_ and thus formed a miscible system. However, the T_g_ values of the pure polymers were in close proximity of one another: 62.5 (±0.5) °C for PEtOx and 57.0 (±0.1) °C for PsecBuOx in the second heating cycle. This means that if immiscibility would occur, the two T_g_s could be in each other’s vicinity and would thus appear as one T_g_ in the thermogram as previously discussed by Bosma et al. [41] and by Jorda and Wikes [42]. To exclude this risk of misinterpretation, solid-state ^1^H-wideline relaxation NMR measurements were carried out on solvent-casted films of PEtOx/PsecBuOx to determine the T_1H_ and T_1ρH_ values of the pure components and the blends (Table 2). Regarding the T_1H_ relaxation times, a single ‘molar proton fraction averaged’ decay time was observed for the blends, indicating that the blends were indeed miscible. The T_1ρH_ experiment revealed that pure polymers as well as the blends demonstrated a bi-exponential behaviour with a fast (T_1ρH_^S^) and slow decaying part (T_1__ρ__H_^L^). It is clear that the protons in the blends relax strongly via the PsecBuOx, which in this case appeared to be the most efficient relaxation pathway. This means that for the three studied blends, PEtOx and PsecBuOx were homogeneously mixed on a length scale of a few nanometres.

The fact that PEtOx/PPrOx formed an immiscible system based on mDSC while PEtOx/PsecBuOx resulted in a miscible system according to mDSC and ssNMR seems to be counterintuitive as the three polymers are structurally almost identical, apart from their side chain. According to the Flory-Huggins theory [43,44], the free energy of mixing (Δ*G*_mix_) and therefore the miscibility of a polymer blend, can be described by the following equation:

$$\frac{∆G_{mix}\;}{RT}=\frac{\phi_{1}}{V_{1}}\ln\phi_{1}+\frac{\phi_{2}}{V_{2}}\ln\phi_{2}+\chi_{12}\phi_{1}\phi_{2}$$

where $\phi_{i}$ is the volume fraction of polymer i,$\;V_{i}$ the molar volume of polymer i and $\chi_{12}$ the Flory-Huggins interaction parameter. The first two terms describe the combinatorial entropy of polymer mixing and are expected to have a small contribution to $∆G_{mix}$ since both $V_{1}$ and $V_{2}$ will be large. The third and most determining factor is the enthalpy of mixing. A possible hypothesis to explain the miscibility behaviour of the two blends that were investigated here is the fact the PPrOx polymer contains a linear side-chain, while PsecBuOx has a branched side-chain. In the polymer blend of PEtOx and PPrOx, these linear side-chains of PPrOx will allow an efficient stacking of PPrOx molecules, leading to Van der Waals interactions. It might therefore be that PPrOx-PPrOx interactions will be thermodynamically more favourable than PEtOx-PPrOx interactions, leading to an immiscible blend. For PsecBuOx, it is expected that there will be less interactions between PsecBuOx molecules due to steric hindrance of the branched side-chain as well as irregularities coming from the side-chains that consist of a racemic mixture of both enantiomers and that interactions between PEtOx and PsecBuOx will be more favourable in this case.

### 2.3. Solid State Analysis of Binary and Ternary Amorphous Solid Dispersions

**Figure 8:**
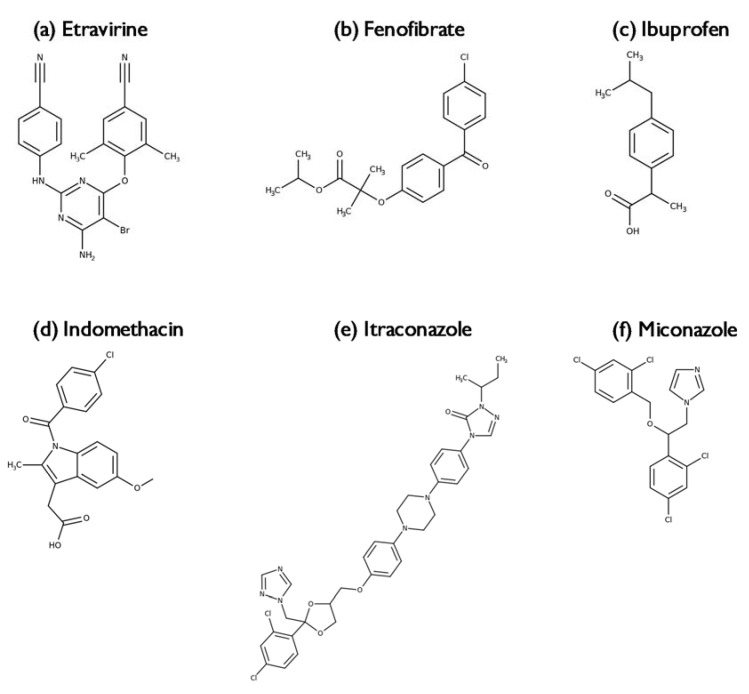
Chemical structures of (**a**) etravirine, (**b**) fenofibrate, (**c**) ibuprofen, (**d**) indomethacin, (**e**) itraconazole and (**f**) miconazole. Created with ChemDraw Professional software (PerkinElmer).

Binary and ternary ASDs were prepared of the PAOx and each of the six model APIs with different physicochemical properties (see Figure 8 and Supplementary Materials S3). In the case of PEtOx/PPrOx where immiscibility was detected with mDSC, it was expected that if the drug would have a preference for one of the two phases, it would cause a more pronounced shift in the T_g_ for that phase and no remarkable shift for the T_g_ of the other phase.

#### 2.3.1. Indomethacin

T_g_s of the binary ASDs that contain IND are listed in Table 3. For the formulations with PEtOx, the high amount of solvent (2.2–1.3%), which evaporated in the same temperature region as where the glass transition occurred, led to a high variation in the measured T_g_ values (see Supplementary Materials S4). This excess of solvent was removed by applying an isothermal segment of 40 °C for 30 min prior to the start of the measurement. For all three polymers a single phase homogeneous ASD was obtained and no melting peaks could be detected. In the case of PEtOx and PsecBuOx, increasing the drug loading (DL) from 10% to 40% IND (*m*/*m*) caused a small decrease in T_g_ of respectively 3.4 °C and 2.8 °C, based on the second heating cycle. For PPrOx, the opposite phenomenon was observed as the T_g_ was 1.3 °C higher for the formulation containing 40% IND compared to the one consisting of 10% IND. These observations can be attributed to the T_g_ of pure amorphous IND, which is situated at 42 °C being higher than the T_g_ of PPrOx and lower than the T_g_s of PEtOx and PsecBuOx [45].

**Figure 9:**
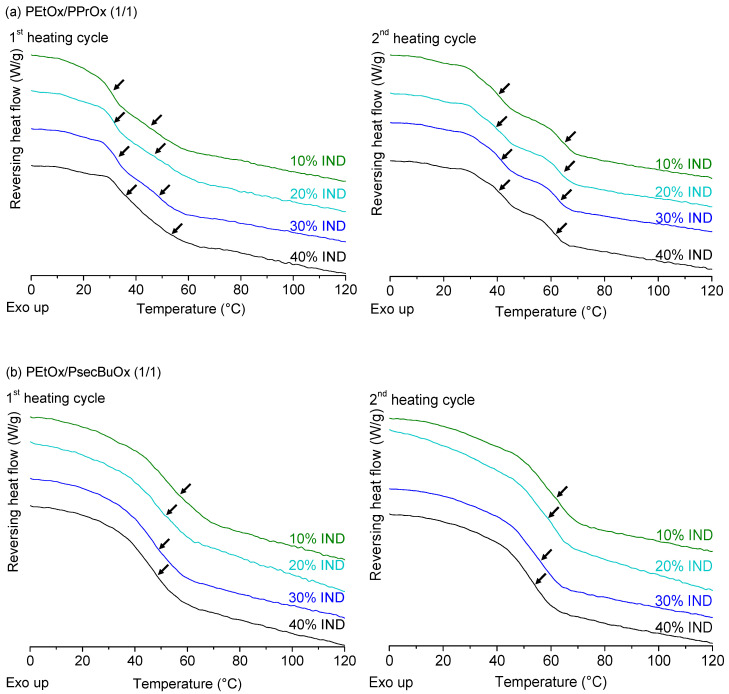
mDSC thermograms of IND, spray dried with a polymer blend of PEtOx/PPrOx (1/1) (**a**) and of PEtOx/PsecBuOx (1/1) (**b**). ASDs with four different drug loadings were prepared. Glass transitions are indicated with arrows in both first (left) and second heating cycle (right). Average T_g_ values (*n* = 3) can be found in Supplementary Materials S5a. Created with Origin 8.5 from OriginLab (Northampton, MA, USA).

For the ternary ASDs of IND with PEtOx/PPrOx (1/1), immiscibility could again be detected in the first heating cycle for all four formulations and was again even more visible during the second heating step (Figure 9a). Due to the fact that the T_g_s of the two polymers were situated close to each other, the signals of the two T_g_s were overlapping and peak deconvolution was performed on the derivative of the reversing heat flow to determine both inflection points. The effect of the increasing drug loading on the position of both T_g_s was difficult to detect and little to no differences were observed between all four formulations, meaning that IND did not seem to have a preference for the PEtOx-rich phase nor the PPrOx-rich phase. However, caution is recommended when interpreting these results, since the T_g_ of pure IND is situated in between the T_g_s of both polymers and could therefore lead to small shifts that could not be observed with mDSC. For the combination of PEtOx and PsecBuOx, a single T_g_ was detected with mDSC for all four DL of IND (Figure 9b). Similar as for the binary ASDs of PEtOx and PsecBuOx, a decrease in T_g_ was again observed with increasing DL.

#### 2.3.2. Itraconazole

For ITZ, binary ASDs based on PEtOx were completely amorphous and showed a single T_g_ (Table 4). Similar observations were done for ASDs of PPrOx and PsecBuOx with 10% and 20% ITZ. Formulations of the latter two respective polymers with a 30 or 40% DL on the other hand had an exothermic event in their thermograms due to cold crystallization and this event was followed by a melting endotherm of ITZ (see Supplementary Materials S6a). For the ASDs of PPrOx, the onset of crystallization was located at 114.9 (±2.1) °C and 112.4 (±0.9) °C for 30% and 40% DL, while for PsecBuOx the onset temperature was at 121 (±2.1) °C and 122 (±0.6) °C for the respective drug loadings. The areas under the curve of the crystallization peaks were equal to the areas of the melting peaks meaning that the sample was originally completely amorphous or only a very small, undetectable amount of crystalline material was present. In the second heating cycle no cold crystallization or melting was detected for these ASDs. In the case of PEtOx-based ASDs there was a small increase in T_g_, measured in the first heating cycle, when more ITZ was present in the formulation. The T_g_s that were recorded in the second heating cycle, after the removal of solvent and water, had almost similar values for the four different drug loadings. This can be attributed to the fact that the T_g_ of pure amorphous itraconazole is 59 °C, which is extremely close to the T_g_ of PEtOx [46]. For the ASDs composed of PPrOx or PsecBuOx on the other hand, an increase in the T_g_ of the second heating cycle was observed when the drug loading was increased from 10% to 40% ITZ as the T_g_ of ITZ is higher.

**Figure 10:**
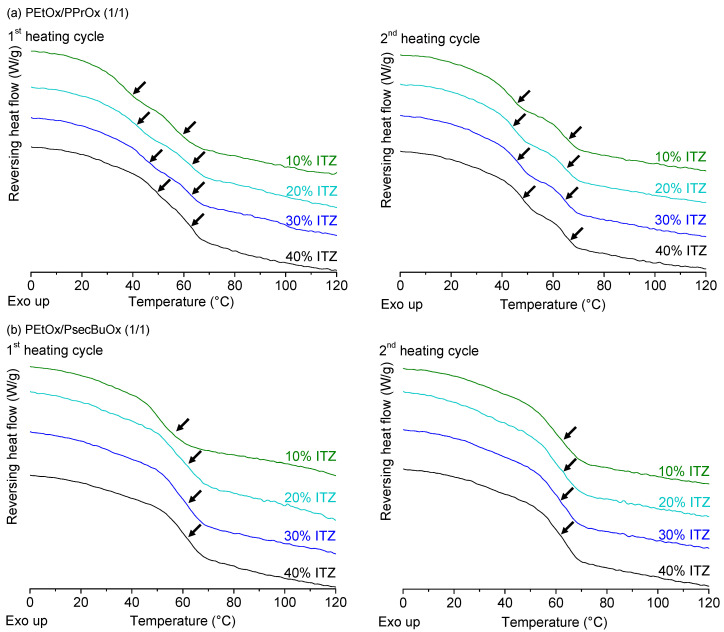
mDSC thermograms of ITZ, spray dried with a polymer blend of PEtOx/PPrOx (1/1) (**a**) and of PEtOx/PsecBuOx (1/1) (**b**). ASDs with four different drug loadings were prepared. Glass transitions are indicated with arrows in both first (left) and second heating cycle (right). Average T_g_ values (*n* = 3) can be found in Supplementary Materials S5b. Created with Origin 8.5 from OriginLab (Northampton, MA, USA).

For the ternary ASDs of ITZ with PEtOx/PPrOx (1/1) and with PEtOx/PsecBuOx (1/1), similar phenomena were observed concerning the miscibility of the polymers as for the ASDs with IND (Figure 10). PEtOx/PPrOx ASDs of ITZ clearly had two distinct T_g_s, while PEtOx/PsecBuOx ASDs showed only one T_g_. Similarly to the PEtOx/PPrOx ASDs of IND, it was difficult to conclude whether ITZ was evenly distributed over PEtOx and PPrOx due to the close proximity of the drug’s T_g_ (59 °C) and the T_g_ of the polymers [46]. For both polymer blends, cold crystallization, followed by melting, was now only observed for the formulation containing 40% ITZ (see Supplementary Materials S6b). This indicates that ITZ was less prone to crystallization upon heating in the DSC when formulated with the polymer blends than with the single polymers PPrOx and PsecBuOx as a binary ASD. A similar observation was done by Yang et al. for immiscible blends of polyvinylpyrrolidone vinyl acetate (PVP-VA) and Eudragit E PO with felodipine [32]. In their work, ternary ASDs were less susceptible to crystallization at 40 °C compared to the binary formulations. The authors attributed this improved stability to the fact that the hydrophobic Eudragit E PO made the ternary ASD less hygroscopic, while the hydrophilic PVP-VA was believed to improve the drug’s miscibility through drug-polymer interactions and the increased miscibility of the drug in the matrix [32]. However, in our case, both the miscible and immiscible PAOx blends were less sensitive to crystallization, indicating that immiscibility of the polymer blend might not be a prerequisite to increase the stability of the ASD.

#### 2.3.3. Miconazole, Fenofibrate, Ibuprofen and Etravirine

**Figure 11:**
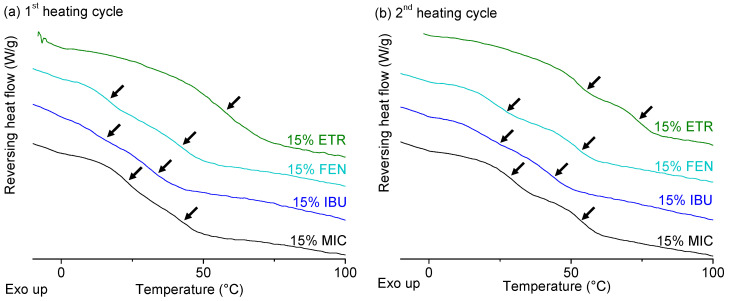
Reversing heat flow, recorded in the first (**a**) and second heating (**b**) cycle of PEtOx/PPrOx (1/1) ASDs with etravirine (ETR), fenofibrate (FEN), ibuprofen (IBU) and miconazole (MIC). The drug loading was fixed at 15% for all four formulations. Glass transitions are indicated with arrows in both first (left) and second heating cycle (right). Created with Origin 8.5 from OriginLab (Northampton, MA, USA).

Based on the mDSC thermograms of ITZ and IND, addition of a poorly water-soluble drug did not seem to impact the phase behaviour of PEtOx/PsecBuOx blends. For the immiscible PEtOx/PPrOx based ASDs however, it was not yet clear whether the model drugs had a preference for one of the two phases. For this reason, four additional poorly water-soluble drugs with a T_g_ far below or above the T_g_s of PEtOx and PPrOx were included in the study: MIC, FEN, IBU and ETR (Figure 8). It is clear from Figure 11 that for all formulations with the four model drugs at a DL of 15% (*m*/*m*), both the T_g_ of the PEtOx-rich phase as well as the T_g_ of the PPrOx-rich phase shifted in the direction of the T_g_ of the pure amorphous drug. This was already a strong indication that the drug was indeed equally divided over both polymer fractions. However, to further quantify the effect of the drug on the relative position of the T_g_s, the ratio of T_g_^PEtOx^ to T_g_^PPrOx^ was calculated for the single polymers, the polymer blend without API and the ASDs with MIC, FEN, IBU and ETR (Table 5). As observed in Table 5, the ratios had the same value for all the formulations in both the first as in the second heating cycle and thus our hypothesis about whether or not the APIs had a preference for one of the two polymer fractions based on the relative positions of the two T_g_s was confirmed. For the ASDs based on ETR, no ratio could be calculated for the first heating cycle as only one, though broad, T_g_ could be detected in the first heating cycle.

### 2.4. Effect of Process Conditions on Miscibility

**Figure 12:**
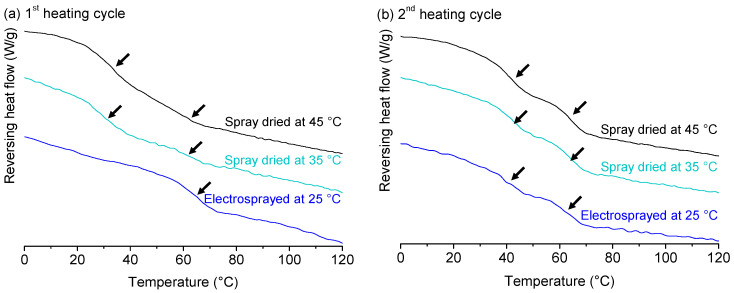
Reversing heat flow, recorded in the first (**a**) and second heating (**b**) cycle of PEtOx/PPrOx (1/1), manufactured via spray drying at 45 °C, at 35 °C and electrospraying at 25 °C. Glass transitions are indicated with arrows in both first (left) and second heating cycle (right). Created with Origin 8.5 from OriginLab (Northampton, MA, USA).

Since it is known that temperature and processing method can have an important effect on the miscibility behaviour of a polymer blend [24], both PEtOx/PPrOx (1/1) and PEtOx/PsecBuOx (1/1) were processed at three different temperatures: 45 °C (spray drying), 35 °C (spray drying) and 25 °C (electrospraying). Electrospraying was selected as a processing method since it allowed efficient solvent evaporation at 25 °C, which could not be achieved via spray drying. The mDSC thermograms of PEtOx/PPrOx (1/1) for all three process conditions are shown in Figure 12. Both spray dried samples clearly showed two T_g_s in the first heating cycle (Figure 12a), meaning that at process conditions of 45 °C and 35 °C an immiscible blend was formed with spray drying. However, the sample that was electrosprayed at 25 °C appeared to be miscible since only one T_g_ was detected during the first heating cycle, which may be a result of the lower processing temperature, the faster solvent evaporation during electrospraying, the presence of residual solvent (1.27%) or a combination of these factors. During the second heating cycle (Figure 12b), immiscibility was more pronounced in the spray dried samples as the two T_g_s were even more clear. Despite the fact that the electrosprayed PEtOx/PPrOx (1/1) was initially a miscible system when formulated at a temperature of 25 °C, two T_g_s were observed during the second heating cycle, indicating phase separation. Based on this observation, it could be concluded that the initially single-phase PEtOx-PPrOx (1-1) electrosprayed sample was not a thermodynamically stable system. It was not possible to draw any conclusions based on relaxation times, recorded with ssNMR, since the relaxation times of the pure PEtOx and PPrOx were too close to one another, as discussed above. The polymer blend of PEtOx and PsecBuOx (1/1) always resulted in a miscible blend, regardless of process conditions that were applied (see Supplementary Materials S7).

## 3. Materials and Methods

### 3.1. Materials

2-Ethyl-2-oxazoline was obtained from Polymer Chemistry Innovations (Tucson, AZ, USA). All other chemicals that were used for the synthesis of the polymers (Section 3.2 Preparation of polymers) were obtained from Sigma-Aldrich (Overijse, Belgium). Fenofibrate was purchased from Hangzhou Apichem Technology CO LTD (Hangzhou, China), ibuprofen from CERTA n.v., Braine-l’Alleud, Belgium and indomethacin (PubChem CID: 3715) was obtained from ThermoFisher (Kandel, Germany). Etravirine, itraconazole and miconazole were a gift sample from Janssen Pharmaceutica NV (Beerse, Belgium). Dichloromethane (DCM) was purchased from Fisher Scientific (Loughborough, UK), acetone from Chemlab (Zedelgem, Belgium) and phosphorus pentoxide from ACROS Belgium (Geel, Belgium). All materials were used as received.

### 3.2. Preparation of Polymers

Poly(2-ethyl-2-oxazoline), poly(2-*n*-propyl-2-oxazoline) and poly(2-*sec*-butyl-2-oxazoline) were prepared following our recently developed protocol for making high molar mass PAOx as described in reference [47]. 2-Ethyl-2-oxazoline (EtOx; Polymer Chemistry Innovations) was purified via fractional distillation and purification over barium oxide. 2-*n*-Propyl-2-oxazoline (nPrOx) and 2-*sec*-butyl-2-oxazoline (secButOx) were synthesized via the Witte-Seeliger method from their corresponding nitriles, i.e., butyronitrile and 2-methylbutyronitrile, respectively [48]. The purification of nPrOx and secButOx was carried out similarly to that of EtOx. Finally, an additional distillation after drying over molten sodium was applied. The polymerizations were carried out in chlorobenzene (PhCI) as solvent, which was purified via consecutive washing steps with concentrated H_2_SO_4_, saturated NaHCO_3_(aq) and water. Drying was done over magnesium sulphate and final drying over CaH_2_. Afterwards a fractional distillation was performed. 2-Phenyl-2-oxazolinium tetrafluoroborate (HPhOx-BF_4_) salt, synthesized following a literature procedure [47], was used as initiator for the polymerization. All polymers were synthesized with a target molar mass of 50,000 g/mol at 60 °C.

Polymer characterization was performed by size-exclusion chromatography (SEC) on an Agilent (Machelen, Belgium) 1260-series HPLC system equipped with a 1260 online degasser, a 1260 ISO-pump, a 1260 automatic liquid sampler (ALS), a thermo-stated column compartment (TCC) at 50 °C equipped with two PLgel 5 pm mixed-D columns (Agilent, Machelen, Belgium) in series, a 1260 diode array detector (DAD) and a 1260 refractive index detector (RID) as well as a Wyatt (Dernback, Germany) TREOS multi-angle light scattering (MALS) detector. The used eluent was *N*,*N*-dimethylacetamide (DMA) containing 50 mM of lithium chloride at an optimized flow rate of 0.5 mL/min. The spectra were analysed using the Agilent ChemStation software with the GPC add on. Number average molecular weight (M_n_) and dispersity (Đ) values were calculated against polymethylmethacrylate (PMMA) molar mass standards from PSS (Mainz, Germany).

PEtOx: SEC with RI detector: M_n,RI_ = 61.4 kg/mol; Đ_RI_ = 1.15 vs PMMA standards.
  SEC with MALS detector: M_n,MALS_ = 40.6 kg/mol; Đ_RI_ = 1.07.
PPrOx: SEC with RI detector: M_n,RI_ = 19.1 kg/mol; Đ_RI_ = 1.16 vs PMMA standards.
  SEC with MALS detector: M_n,MALS_ = 49.6 kg/mol; Đ_RI_ = 1.05.
PsecBuOx: SEC with RI detector: M_n,RI_ = 26.9 kg/mol; Đ_RI_ = 1.22 vs PMMA standards.
  SEC with MALS detector: M_n,MALS_ = 65.9 kg/mol; Đ_RI_ = 1.11.

### 3.3. Preparation of Polymer Blends, Binary and Ternary Amorphous Solid Dispersions by Spray Drying

Polymer blends, binary and ternary ASD formulations were prepared through spray drying. First, the three polymers were spray dried separately to investigate the effect of spray drying on the thermal analysis of the pure polymers. After that, miscibility of the two polymer blends (PEtOx/PPrOx and PEtOx/PsecBuOx) was investigated in the absence of the model APIs. For the combination PEtOx/PPrOx, ratios of 1/3, 1/1 and 3/1 (*m*/*m*) were prepared. In the case of PEtOx/PsecBuOx, miscibility of the two polymers without the presence of an API was investigated at ratios of 2/1, 1/1 and 1/2 (*m*/*m*) through film casting as discussed in Section 3.5 due to low yields with spray drying and a limited amount of polymer. After the assessment of the miscibility of the two blends, binary ASDs of PEtOx, PPrOx and PsecBuOx with IND and ITR were formulated. Four different drug loadings (DLs) were investigated: 10, 20, 30 and 40% (% *m*/*m*). This was followed by the preparation of ternary ASDs of PEtOx/PPrOx (1/1, *m*/*m*) and PEtOx/PsecBuOx (1/1, *m*/*m*) with ITZ and IND at the same four DLs. Next to that, ternary ASDs of ETR, FEN, IBU and MIC in combination with PEtOx/PPrOx (1/1, *m*/*m*) were produced. For these formulations the DL was set at 15% (% *m*/*m*). In all ternary ASDs, polymers were present in a 1/1 (*m*/*m*) ratio.

Except for ETR, all model compounds as well as PAOx polymers could be dissolved in DCM, reaching a solid content of 10% (% *m*/*V*). In the case of formulations with ETR, a solvent mixture of DCM:acetone (1/1, *V*/*V*) was used with a solid content of 2.5% (% *m*/*V*). A lab-scale spray dryer (Buchi mini spray dryer B-190, Flawil, Switzerland) with the following settings was used to prepare the formulations: feed rate of 10 mL/min, inlet temperature of 45 °C, drying air flow rate of 33 m^3^/hour and atomization air flow rate was fixed at 10 L/min. After spray drying, the powder was dried for four consecutive days in a vacuum oven (Mazzali Systems, Monza, Italy) at room temperature and after this secondary drying step, ASDs were stored in the presence of phosphorus pentoxide at −28 °C until further analysis.

### 3.4. Electrospraying

Electrosprayed samples of PEtOx/PPrOx and PEtOx/PsecBuOx in a 1/1 ratio were prepared at 25 °C and at 30% relative humidity (RH) with a climate controlled electrospinning apparatus (EC-CLI, IME Technologies, Geldrop, The Netherlands). For both combinations, solutions with a 1% solid content were prepared by dissolving the polymers in DCM. The feed rate of the solution was set at 0.5 mL/hour, the tip-to-collector distance at 7 or 9 cm and the voltage at 20 kV. Afterwards, the electrosprayed samples were dried in a vacuum oven and stored under the same conditions as discussed here above for the spray dried samples.

### 3.5. Film Casting by Fast Evaporation of the Solvent

The miscibility of PEtOx and PsecBuOx was investigated by preparing films of the two polymers in different ratios (1/2, 1/1 and 2/1, *m*/*m*) and of the individual polymers. PEtOx and PsecBuOx were dissolved in DCM, attaining a solid content of 10%, after which the solvent was then rapidly removed with a Büchi Rotovap R210 (Flawil, Switzerland) at a temperature of 35 °C (Büchi Heating Bad, Flawil, Switzerland). The collected product was subjected to a secondary drying step of four days in a vacuum oven (Mazzali Systems, Monza, Italy) at room temperature prior to modulated differential scanning calorimetry (mDSC) and solid-state nuclear magnetic resonance spectroscopy measurements (ssNMR).

### 3.6. X-ray Powder Diffraction (XRPD)

Solid state of the spray dried formulations was investigated with an X’pert PRO diffractometer (PANalytical, Almelo, The Netherlands), equipped with a Cu tube (Kα λ = 1.5418 Å) and a generator set-up at 45 kV and 40 mA. All X-ray powder diffraction (XRPD) measurement were performed in the transmission mode at room temperature by fixating the sample between Kapton^®^ Polyimide Thin-films (PANalytical, USA). Samples were scanned from 4 to 40° 2θ with 400 s counting time and 0.0167° step size. Next to those measurements, one additional temperature resolved experiment was executed for spray-dried PPrOx at a temperature of 25–155 °C with a heating increment of 10 °C between each measurement. Diffractograms of both experiments were processed with X’Pert Data Viewer (Version 1.7, PANalytical, Almelo, The Netherlands).

### 3.7. Modulated Differential Scanning Calorimetry (mDSC)

Phase behaviour and miscibility of the formulations was investigated with mDSC using a Q2000 and Discovery 2500 DSC (TA Instruments, Leatherhead, U.K.). Both systems were equipped with a refrigerated cooling system (RCS90) and dry nitrogen was used as a purge gas with a flow rate of 50 mL/min. Temperature and enthalpy calibration was performed with indium. In addition, temperature calibrations were also performed for octadecane and tin. Finally, the equipment was also calibrated for heat capacity with sapphire.

For mDSC measurements, 2–4 mg of sample was accurately weighed into standard aluminium pans (TA instruments, Zellik, Belgium) and crimpled with a standard lid (TA instruments, Zellik, Belgium). For all measurements, modulation parameters were set at a modulation amplitude of 0.212 °C and a period of 40 s. All samples were subjected to a heat-cool-heat procedure. Samples with no model API, IND and ITZ, were heated from −10 to 180 °C at a heating rate of 2 °C/min, cooled to −10 °C at 20 °C/min and finally heated again at 2 °C/min to 180 °C. Samples that contained ETR were heated to 220 °C instead of 180 °C, while ASDs consisting of IBU, FEN and MIC were heated from −50 or −40 to 110 °C. Heating and cooling rates remained the same for all samples. Except for the electrosprayed samples where the yield was limited, all measurements were carried out in triplicate. Collected mDSC thermograms were analysed with Universal Analysis (version 4.5A, TA Instruments, Leatherhead, UK) and TRIOS software (version 4.4, TA Instruments, Leatherhead, UK). Peak deconvolutions of the derivative of the reversing heat flow were determined in Origin 8.5 (OriginLab, Northampton, MA, USA). For all samples, glass transition temperatures were calculated based on the inflection point in the reversing heat flow.

### 3.8. Thermogravimetric Analysis (TGA)

The amount of residual solvent and water in the formulations was determined by thermogravimetric analysis using a SDT Q600 TGA (TA-Instruments, Leatherhead, UK). Weight loss due to evaporation of solvent was monitored while heating the sample from room temperature to 130 °C at a rate of 5 °C/min while being exposed to air. The difference in mass was calculated with Universal Analysis software (version 4.5A, TA Instruments, Leatherhead, UK).

### 3.9. Solid-State Nuclear Magnetic Resonance (ssNMR)

Solid-state ^1^H-wideline NMR measurements were carried out at ambient temperature on a Jeol 600 spectrometer in a dedicated wide-line probe equipped with a 3 mm coil using the solid echo technique (90°_x’_ - t_se_ - 90°_y’_ - t_se_ - acquire) to overcome the effect of the dead-time of the receiver. The 90° pulse length was set to 1.9 µs and spectra were recorded with a spectral width of 3.125 MHz (0.32 µs dwell time) allowing an accurate determination of the echo maximum. The echo maximum was set to time zero. The samples were placed in zirconia tubes, which were closed with Kel-F stoppers.

The T_1H_ relaxation decay times (spin-lattice relaxation in the lab frame) were measured by placing an inversion recovery filter in front of the solid echo part (180°_x′_ - t - 90°_x′_ - t_se_ - 90°_y′_ - t_se_ - acquire). The integrated proton signal intensity was analysed monoexponentially as a function of the variable inversion time t according to:

$$I\left(t\right)=I_{0}\left[1-2\exp\left(\frac{-t}{T_{1H}}\right)\right]+c^{te}$$

The *T*_1ρ*H*_ decay times (spin-lattice relaxation in the rotating frame) were measured by applying a spin-lock field (50 kHz) of variable duration, t, after the initial 90°_x′_ pulse in the solid echo pulse sequence (90°_x′_ - t - t_se_ - 90°_y′_ - t_se_ - acquire). The integrated proton signal intensity was analysed biexponentially as a function of the variable duration of the spin-lock field t according to the equation:

$$I\left(t\right)=I_{0}{}^{S}exp\left(\frac{-t}{T_{1\rho H}{}^{S}}\right)+I_{0}{}^{L}exp\left(\frac{-t}{T_{1\rho H}{}^{L}}\right)+c^{te}$$

All experimental data were analysed using a non-linear least-squares fit (Levenberg-Marquardt algorithm). A preparation delay of 5× the longest T_1H_ relaxation decay time was always respected between successive accumulations to obtain quantitative results.

## 4. Conclusions

In the present work, miscibility of water-soluble (PEtOx) with a water-insoluble PAOx (PPrOx or PsecBuOx) was evaluated as the basis for future development of a potential alternative carrier system for ASDs with a delayed drug release to prevent the risk for precipitation upon drug release. In the case of spray dried PEtOx-PPrOx, immiscibility was already detected in the first heating cycle for the 1-to-1 ratio of the polymer blend, while the blend containing 75% PEtOx appeared to be heterogeneous as one of the three mDSC thermograms showed two T_g_ signals. The immiscibility became even more pronounced in the second heating cycle, indicating that manufacturing processes such as hot melt extrusion are expected to affect the phase behaviour of this polymer blend. When ternary ASDs with poorly water-soluble drugs were prepared, the blend remained immiscible and the tested drugs were equally distributed over both phases. The temperature and processing method appeared to have an effect on the miscibility of PEtOx-PPrOx, since the electrosprayed sample that was processed at 25 °C, appeared initially miscible. However, this was not a thermodynamically stable system as two T_g_s appeared during the second heating cycle, indicating that high temperature induced phase separation.

For the blend with PEtOx and PsecBuOx, it was difficult to unambiguously determine if the polymers were indeed miscible since the T_g_s of the pure polymers were within each other’s vicinity. ssNMR was needed as a complementary analysis technique to confirm that the polymers were miscible based on their T_1H_ and T_1ρH_ relaxation times. Addition of the drug did not affect the miscibility of the polymers and a single T_g_ was observed for both IND and ITZ at different drug loadings.

This work highlights the potential of PAOx as a novel polymer class for ASDs. Despite the fact that the three polymers were structurally very similar, one combination led to an immiscible system while the other blend resulted in a miscible system. A possible explanation for this miscibility behaviour was the presence of branched, racemic chiral side chains in the PsecBuOx polymer, leading to favourable mixing behaviour with PEtOx and its absence in the PPrOx side-chain. It is clear that as various functional groups can easily be incorporated as a side chain, PAOx can serve as a formulation platform to design tailor-made carriers with specific solution, thermal, mechanical and also miscibility behaviour.

## Figures and Tables

**Table 1:** Spin lattice relaxation times recorded in a lab frame (T_1_) and the rotating frame (T_1ρ_) of spray dried PEtOx, PPrOx and a 1-1 (*m*/*m*) combination of both polymers. In the rotating frame, a short (S) and long (L) relaxation time was detected for all three samples. I_0_ gives the percentages of both relaxation times.

Sample (*m*/*m*)	T_1H_ (s)	T_1ρH_ (ms)			
		T_1ρH_^S^	I_0_^S^ (%)	T_1ρH_^L^	I_0_^L^ (%)
PEtOx	2.3	0.5	18.5	6.9	81.5
PPrOx	1.5	1.0	19.1	7.6	80.9
PEtOx/PPrOx (1/1)	1.7	0.6	17.9	6.1	82.1

**Table 2:** Spin lattice relaxation times recorded with a lab frame (T_1_) and a rotating frame (T_1ρ_) of PEtOx, PsecBuOx and combinations of the two PAOx: 2/1, 1/1 and 1/2 (*m*/*m*). With the rotating frame, a short (S) and long (L) relaxation time was detected for all five samples. I_0_ gives the percentages of both relaxation times.

Sample (*m*/*m*)	T_1H_ (s)	T_1ρH_ (ms)			
		T_1ρH_^S^	I_0_^S^ (%)	T_1ρH_^L^	I_0_^L^ (%)
PEtOx	1.74	0.9	14.7	7.0	85.3
PsecBuOx	0.78	1.3	9.4	8.9	90.6
PEtOx/PsecBuOx (2/1)	1.28	1.5	10.0	9.3	90.0
PEtOx/PsecBuOx (1/1)	1.10	1.9	12.0	9.7	88.0
PEtOx/PsecBuOx (1/2)	0.97	1.6	11.0	9.6	89.0

**Table 3:** Average T_g_s (± standard deviation) of binary ASDs with IND and PEtOx, PPrOx or PsecBuOx. mDSC measurements were performed in triplicate. Drug loading was increased from 10% to 40% IND.

Drug Loading	1st Heating Cycle (°C)			2nd Heating Cycle (°C)		
(% *m*/*m*)	PEtOx *	PPrOx	PsecBuOx	PEtOx *	PPrOx	PsecBuOx
10% IND	62.1 (±0.3)	31.2 (±2.0)	49.0 (±0.7)	64.9 (±0.7)	39.9 (±0.5)	55.2 (±0.6)
20% IND	63.5 (±1.4)	32.8 (±0.2)	47.8 (±0.9)	63.7 (±0.3)	40.6 (±0.5)	53.7 (±0.6)
30% IND	60.6 (±0.3)	33.5 (±0.3)	49.6 (±0.7)	62.4 (±0.5)	41.5 (±0.8)	52.6 (±0.2)
40% IND	59.6 (±0.5)	32.7 (±2.3)	49.5 (±1.5)	61.5 (±0.2)	41.2 (±0.6)	52.4 (±0.3)

* An additional isothermal segment was performed before the start of the first heating cycle for spray dried PEtOx to remove the excess of solvent and water, present in the formulation.

**Table 4:** Average T_g_s (± standard deviation) of binary ASDs with ITZ and PEtOx, PPrOx or PsecBuOx. mDSC measurements were performed in triplicate. Drug loading was increased from 10% to 40% IND.

Drug Loading	1st Heating Cycle (°C)			2nd Heating Cycle (°C)		
(% *m*/*m*)	PEtOx	PPrOx	PsecBuOx	PEtOx	PPrOx	PsecBuOx
10% ITZ	56.3 (±1.6)	32.7 (±0.9)	55.6 (±1.2)	64.7 (±0.8)	42.8 (±0.1)	56.8 (±0.3)
20% ITZ	58.9 (±2.0)	39.2 (±0.9)	53.8 (±0.7)	65.3 (±0.4)	44.0 (±0.2)	57.1 (±0.9)
30% ITZ	59.8 (±0.8)	41.4 (±0.2)	56.7 (±1.1)	64.6 (±0.2)	46.2 (±0.6)	57.8 (±0.9)
40% ITZ	60.4 (±0.8)	43.6 (±0.5)	56.8 (±0.6)	65.2 (±0.2)	47.9 (±0.4)	59.0 (±0.3)

**Table 5:** T_g_s of PEtOx, PPrOx, PEtOx/PPrOx (1/1) without drug and the respective polymer blend with 15% etravirine (ETR), 15% fenofibrate (FEN), 15% ibuprofen (IBU) and 15% miconazole (MIC). The ratio of the two T_g_s was calculated for each formulation.

Drug Loading	1st Heating Cycle (K)			2nd Heating Cycle (K)		
(% *m*/*m*)	T_g_^PPrOx^	T_g_^PEtOx^	$\frac{T_{g}^{PEtOx}}{T_{g}^{PPrOx}}$	T_g_^PPrOx^	T_g_^PEtOx^	$\frac{T_{g}^{PEtOx}}{T_{g}^{PPrOx}}$
PEtOx spray dried	/	336.5 (±0.4) *	1.10	/	336.6 (±0.1) *	1.07
PPrOx spray dried	305.0 (±2.2)	/		314.4 (±1.1)	/	
Spray dried without drug	306.1 (±0.1)	330.7 (±1.9)	1.08	317.0 (±0.9)	336.4 (±0.5)	1.06
15% ETR	333.0 (±4.17)		/	325.3 (±0.2)	347.3 (±0.0)	1.07
15% FEN	291.3 (±0.1)	313.5 (±1.2)	1.08	297.3 (±0.2)	323.1 (±0.4)	1.09
15% IBU	287.9 (±0.8)	305.3 (±0.6)	1.06	295.4 (±0.6)	314.5 (±0.4)	1.06
15% MIC	295.9 (±2.0)	317.3 (±2.9)	1.07	302.2 (±1.8)	326.5 (±0.7)	1.08

* T_g_ determined after an isothermal segment was applied to remove the excess of the residual solvent.

## Supplementary Materials

The following are available online: Supplementary Materials S1: Glass transition temperatures of raw material and spray dried PAOx; S2: Glass transition temperatures of PEtOx/PPrOx and PEtOx/PsecBuOx polymer blends; S3: Overview of the six model APIs that were investigated for this study and their corresponding physicochemical properties; S4: Effect of residual solvent on the T_g_ of IND-PEtOx ASDs; S5: Glass transition temperatures of ternary amorphous solid dispersions; S6: Cold crystallization and melting in binary and ternary ASDs of ITZ; S7: PEtOx/PsecBuOx (1/1, *m*/*m*) prepared via spray drying at 45 °C, spray drying at 35 °C and electrospraying at 25 °C.
