# Supplementary material for: Unravelling the Miscibility of Poly(2-oxazoline)s: A Novel Polymer Class for the Formulation of Amorphous Solid Dispersions
Source: Molecules. 2020 Aug 6;25(16):3587. doi: 10.3390/molecules25163587 (PMC7465563; doi:10.3390/molecules25163587)
Supplement: Supplementary file 1 [file molecules-25-03587-s001.pdf]

molecules

MDPI

Supplementary Materials

Unravelling the Miscibility of Poly(2-oxazoline)s: A Novel Polymer Class for the Formulation of Amorphous Solid Dispersions

Melissa Everaerts [1], Ali Tigrine [2], Victor R. de la Rosa [2], Richard Hoogenboom [2], Peter Adriaensens [3], Christian Clasen [4] and Guy Van den Mooter [1,*]

[1] Drug Delivery and Disposition, Department of Pharmaceutical and Pharmacological Sciences, KU Leuven, 3000 Leuven, Belgium; melissa.everaerts@kuleuven.be

[2] Supramolecular Chemistry Group, Centre of Macromolecular Chemistry (CMaC), Department of Organic and Macromolecular Chemistry, Ghent University, Krijgslaan 281-S4, 9000 Ghent, Belgium; ali.tigrine@ugent.be (A.T.); victorretamero.delarosa@ugent.be (V.R.D.); richard.hoogenboom@ugent.be (R.H.)

[3] Applied and Analytical Chemistry Department, Institute for Materials Research, Hasselt University, 3590 Diepenbeek, Belgium; peter.adriaensens@uhasselt.be

[4] Department of Chemical Engineering, Soft Matter, Rheology and Technology, KU Leuven, 3001 Leuven, Belgium; christian.clasen@kuleuven.be

* Correspondence: guy.vandenmooter@kuleuven.be; Tel.: +32 16 330304

Academic Editor: Maria Cristina Bonferoni

Received: 8 July 2020; Accepted: 31 July 2020; Published: date

Abstract: Water-soluble polymers are still the most popular carrier for the preparation of amorphous solid dispersions (ASDs). The advantage of this type of carrier, is the fast drug release upon dissolution of the water-soluble polymer and thus the initial high degree of supersaturation of the poorly soluble drug. Nevertheless, the risk for precipitation due to fast drug release is a phenomenon that is frequently observed. In this work, we present an alternative carrier system for ASDs where a water-soluble and water-insoluble carrier are combined to delay the drug release and thus prevent this onset of precipitation. Poly(2-alkyl-2-oxazoline)s were selected as a polymer platform since the solution properties of this polymer class depend on the length of the alkyl sidechain. Poly(2-ethyl-2-oxazoline) (PEtOx) behaves as a water-soluble polymer at body temperature, while poly(2-*n*-propyl-2-oxazoline) (PPrOx) and poly(2-*sec*-butyl-2-oxazoline) (PsecBuOx) are insoluble at body temperature. Since little was known about the polymer's miscibility behavior and especially on how the presence of a poorly-water soluble drug impacted their miscibility, a preformulation study was performed. Formulations were investigated with X-ray powder diffraction, differential scanning calorimetry (DSC) and solid-state nuclear magnetic resonance spectroscopy. PEtOx/PPrOx appeared to form an immiscible blend based on DSC and this was even more pronounced after heating. The six drugs that were tested in this work did not show any preference for one of the two phases. PEtOx/PsecBuOx on the other hand appeared to be miscible forming a homogeneous blend between the two polymers and the drugs.

Keywords: Amorphous solid dispersions; poly(2-oxazoline)s; miscibility; modulated differential scanning calorimetry; solid-state nuclear magnetic resonance spectroscopy

Table S1. Glass transition temperatures of raw material and spray dried PAOx.

Polymer	T_g in 1st heating cycle (°C)		T_g in 2nd heating cycle (°C)	
	Raw material	Spray dried	Raw material	Spray dried
PEtOx	61.8 (±3.7)	63.4 (±0.4)*	64.9 (±0.5)	63.4 (±0.1)*
PPrOx	32.0 (±1.3)	31.9 (±2.2)	42.3 (±0.1)	41.3 (±1.1)
PsecBuOx	49.9 (±0.4)	51.5 (±1.5)	56.5 (±0.3)	55.4 (±0.9)

* An additional isothermal segment was performed before the start of the first heating cycle for spray dried PEtOx to remove the excess of solvent and water, present in the formulation.

Table S2. Glass transition temperatures of PEtOx/PPrOx and PEtOx/PsecBuOx polymer blends.

S2a) Glass transition temperatures of spray dried PEtOx/PPrOx polymer blends (n=3)

PEtOX/PPrOx (m/m)	T_g in 1st heating cycle (°C)		T_g in 2nd heating cycle (°C)	
3/1*	29.7<br>43.1<br>40.5	62.0	41.5 (±2.0)	64.7 (±1.3)
1/1	32.9 (±0.1)	57.5 (±1.9)	43.8 (±0.9)	63.2 (±0.5)
1/3	28.1 (±1.1)		41.5 (±1.0)	64.4 (±2.9)

* Due to the inhomogeneity of the 3/1 (m/m) PEtOx/PPrOx blend, no average T_g could be calclulated for this sample.

S2b) Glass transition temperatures of film-casted PEtOx/PsecBuOx polymer blends (n=3)

PEtOX/PsecBuOx (m/m)	T_g in 1st heating cycle (°C)	T_g in 2nd heating cycle (°C)
2/1	51.8 (±2.0)	62.2 (±1.4)
1/1	56.3 (±1.5)	58.9 (±1.8)
1/2	54.5 (±2.5)	57.7 (±0.7)

Table S3. Overview of the six model APIs that were investigated for this study and their corresponding physicochemical properties.

Compound	Therapeutic Class[a]	Bruto Formula	Molecular weight (g/mol)[a]	Van der Waals Surface Area (3D)[b]	Polar Surface Area (2D)[b]	Log P[b]	T_m (°C)	T_g (°C)
Etravirine	Antiviral agent	$C_{20}H_{15}BrN_6O$	435.285	501.86	120.64	5.54	265[c]	99[f]
Fenofibrate	Antilipemic agent	$C_{20}H_{21}ClO_4$	360.834	533.55	52.60	5.28	79-82[d]	32.4[e]
Ibuprofen	NSAID	$C_{13}H_{18}O_2$	206.285	356.40	37.30	3.84	75-78[d]	-45[g]
Indomethacin	NSAID	$C_{19}H_{16}ClNO_4$	357.79	475.59	68.53	3.53	158-162[d]	42[h]
Itraconazole	Antifungal Agent	$C_{35}H_{38}Cl_2N_8O_4$	705.641	965.40	100.79	7.31	166[e]	59.2[i]
Miconazole	Antifungal Agent	$C_{18}H_{14}Cl_4N_2O$	416.123	490.38	27.05	5.96	83-87[d]	1.6[i]

[a] Information obtained from PubChem; [b] Calculated with MarvinSketch 19,11 (ChemAxon); [c] Decomposition temperature of etravirine. Y. Huang, W.-G. Dai, Acta Pharm. Sin. B. (2014); [d] from European Pharmacopoeia 8.0; [e] T. Pas et al., Int. J. Pharm. (2018); [f] S. Qi et al., J. Pharm. Sci. (2010); [g] E. Dudognon et al., Pharm. Res. (2008); [h] R. Chokshi et al., J. Pharm. Sci. (2008); [i] K. Six et al., Int. J. Pharm. (2001).

Material S4. Effect of residual solvent on the T_g of IND/PEtOx ASDs.

a)

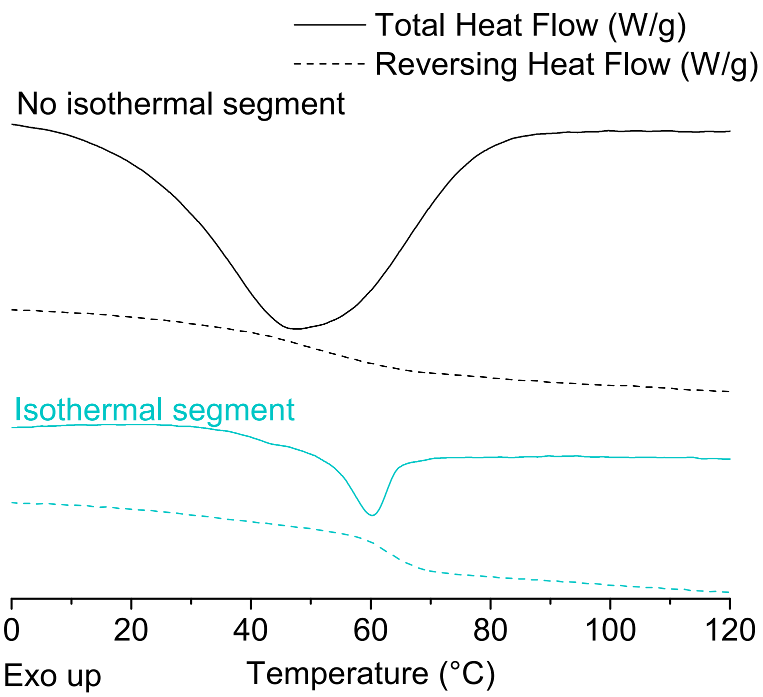

Figure S4. mDSC thermograms of IND/PEtOx ASD (10% DL) with and without an isothermal segment of 40°C for 30 minutes prior to the first heating step.

b) Table S4. Experimental T_g values for IND/PEtOx samples, measured with and without an isothermal segment.

**Drug loading (% m/m)**	**1st heating cycle (°C)**		**2nd heating cycle (°C)**	
	**No isothermal segment**	**Isothermal segment**	**No isothermal segment**	**Isothermal segment**
**10% IND**	57.5 (±8.0)	62.1 (±0.3)	63.8 (±0.8)	64.9 (±0.7)
**20% IND**	58.2 (±6.6)	63.5 (±1.4)	63.8 (±0.3)	63.7 (±0.3)
**30% IND**	53.0 (±2.4)	60.6 (±0.3)	62.4 (±0.6)	62.4 (±0.5)
**40% IND**	56.5 (±1.4)	59.6 (±0.5)	61.4 (±0.6)	61.5 (±0.2)

Table S5. Glass transition temperatures of ternary amorphous solid dispersions.

S5a) Glass transitions temperatures (n=3) of IND ternary ASDs consisting of PEtOx/PPrOx (1/1, m/m) or PEtOx/PsecBuOx (1/1, m/m)

**Drug loading (% m/m)**	**PEtOx/PPrOx (1/1, m/m)**				**PEtOx/PsecBuOx (1/1, m/m)**	
	**1st heating cycle**		**2nd heating cycle**		**1st heating cycle**	**2nd heating cycle**
10% IND	32.4 (±1.0)	50.9 (±3.0)	42.0 (±0.7)	63.5 (±0.6)	50.5 (±1.9)	62.5 (±3.2)
20% IND	32.1 (±1.7)	52.5 (±2.8)	41.6 (±1.6)	63.4 (±1.2)	46.7 (±0.7)	59.4 (±1.8)
30% IND	34.2 (±1.3)	52.0 (±2.6)	41.1 (±0.8)	61.9 (±0.6)	46.1 (±0.7)	57.2 (±1.3)
40% IND	36.4 (±1.4)	50.7 (±3.4)	42.9 (±1.1)	61.2 (±2.1)	44.9 (±2.2)	54.2 (0.9)

S5b) Glass transitions temperatures (n=3) of ITZ ternary ASDs consisting of PEtOx/PPrOx (1/1, m/m) or PEtOx/PsecBuOx (1/1, m/m)

**Drug loading (% m/m)**	**PEtOx/PPrOx (1/1, m/m)**				**PEtOx/PsecBuOx (1/1, m/m)**	
	**1st heating cycle**		**2nd heating cycle**		**1st heating cycle**	**2nd heating cycle**
10% ITZ	36.7 (±0.2)	57.7 (±1.9)	43.9 (±1.1)	64.8 (±1.3)	51.4 (±2.1)	60.2 (±2.8)
20% ITZ	42.2 (±2.4)	62.2 (±2.3)	44.4 (±1.7)	65.5 (±1.2)	55.5 (±1.2)	59.2 (±1.5)
30% ITZ	42.8 (±1.2)	61.0 (±1.3)	45.9 (±1.8)	65.4 (±1.2)	62.2 (±0.7)	62.1 (±2.2)
40% ITZ	49.4 (±0.7)	61.9 (±1.1)	48.8 (±1.1)	64.6 (±0.1)	60.2 (±1.9)	64.6 (1.1)

Figure S6. Cold crystallization and melting in binary and ternary ASDs of ITZ.

a)

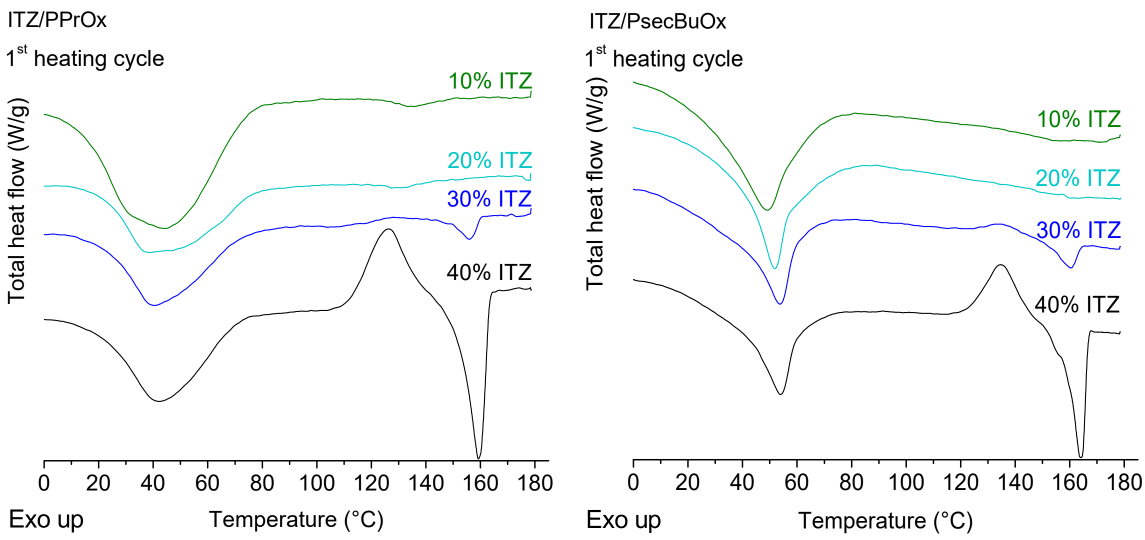

Figure S6a. mDSC thermograms of binary ITZ/PPrOx (left) and ITZ/PsecBuOx (right) where total heat flow is depicted for 10%, 20%, 30% and 40% ITZ (% m/m).

b)

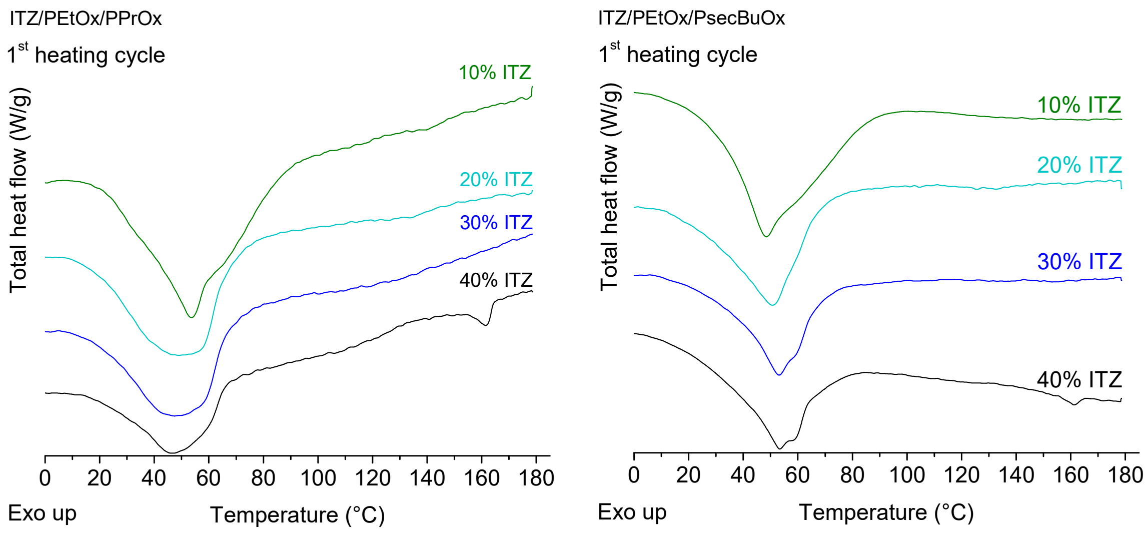

Figure S6b. mDSC thermograms of ternary ITZ/PEtOx/PrOx (left) and ITZ/PEtOx/PsecBuOx (right) where total heat flow is depicted for 10%, 20%, 30% and 40% ITZ (% m/m). The ratio of the polymer blend was fixed at 1/1 ratio (m/m).

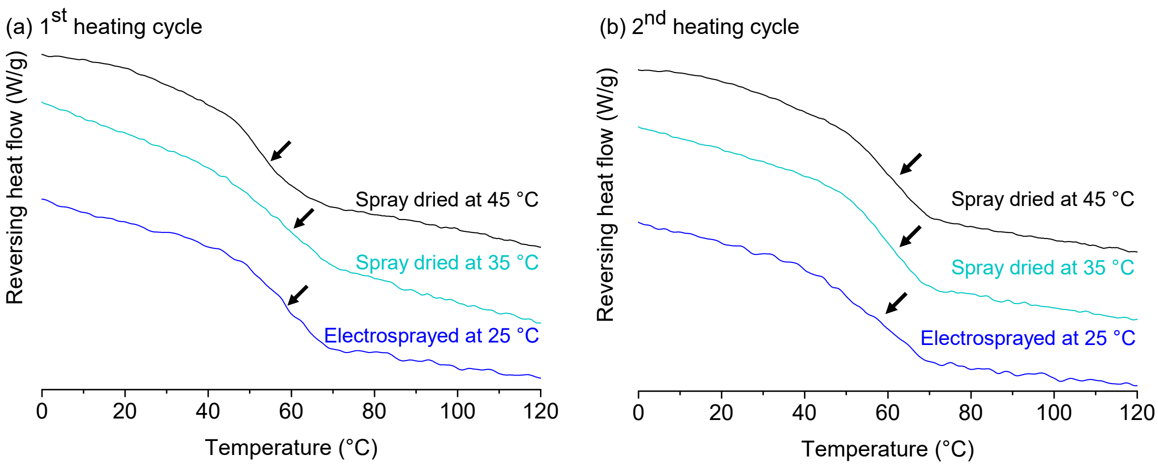

Figure S7. PEtOx/PsecBuOx (1/1, m/m) prepared via spray drying at 45 °C, spray drying at 35 °C and electrospraying at 25 °C.
